# A Post-marketing Surveillance, Single-Centric Study to Evaluate the Safety and Tolerability of VELNEZ as a Space-Occupying Dressing Pack After Ear Surgery

**DOI:** 10.7759/cureus.51732

**Published:** 2024-01-06

**Authors:** Akhil P Singh, Saloni Singh, Ridhima Malik

**Affiliations:** 1 Department of Otolaryngology - Head and Neck Surgery, Sarojini Naidu Medical College, Agra, IND

**Keywords:** velnez, tympanoplasty, myringoplasty, ear pack, ear surgery

## Abstract

Purpose: The purpose of this study was to evaluate the safety and tolerability of VELNEZ (Datt Mediproducts Pvt. Ltd., New Delhi, India) as a space-occupying dressing for controlling hemorrhage after ear surgery.

Method: A total of 21 patients were included in an open-label, interventional, single-arm post-marketing surveillance study to investigate the safety and efficacy of the VELNEZ ear pack. The patients were questioned for collecting data related to the subject’s safety and comfort, adverse events, site assessment, and otoscopic examination from discharge day to last follow-up (eight follow-up visits) at regular intervals. The standardized questionnaires for VELNEZ tolerability (pain/pressure effect, infection, and general satisfaction) were used after ear surgery.

Results: The average hemorrhage control time was 1.08 ± 0.16 minutes. None of the subjects reported moderate pain at any of the study visits following surgery. This biodegradable ear pack had an average disintegration time of 25.4 days in the ear cavity. No postoperative adverse events or serious adverse events were observed.

Conclusion: VELNEZ is safe and effective as a space-occupying dressing pack after ear surgery and is well-tolerated by patients.

## Introduction

Ear surgeries like tympanoplasty and mastoidectomy make up the bulk of surgeries in ENT practice, especially in underdeveloped nations due to poor housing and hygienic conditions. Currently, aside from the graft material for tympanic membrane perforation closure, the use of packing material in the cavity of the middle ear is required in the majority of surgeries. The support material must be safe and biocompatible and should not cause mucosal adhesions, which could result in compromised middle ear ventilation. Ideally, it should be conformable to the ear cavity and support the graft stability long enough for healing [1] and must disintegrate over time leaving no residue.

Several types of support materials are used in various surgical procedures for ear [2]. Non-absorbable packing materials often result in pain following removal and may displace the graft, hence are no longer used. Absorbable ear packs can be manufactured from hyaluronic acid (HA), synthetic, or alternative plant materials. HA-based middle ear packing materials have limited use in otological surgeries because of the physical properties of the fluid nature [3]. However, esterified HA-based packing materials (e.g., MeroGel) with better supporting capacity in comparison to other forms of HA are available. Worldwide, the most commonly used material to provide support to compromised tissues and stability to the tympanic graft after ear surgery is a hemostatic absorbable gelatin sponge (e.g., Gelfoam and Spongostan). However, there are some studies that indicate possible detrimental effects secondary to the usage of Gelfoam [4-6].

VELNEZ (manufactured by Datt Mediproducts Pvt. Ltd., New Delhi, India) is a biodegradable composite that disintegrates within a few days after application. It acts as a space-occupying dressing and tends to improve wound healing as it potentially aids in achieving local hemostasis, minimizes scarring/synechiae, and prevents adhesion by separating the compromised mucosal surfaces. It supports tissue regeneration and helps in the healing process. VELNEZ can be soaked in antibiotic solution and potentially prevent infection when applied after surgery. The purpose of this study is to assess the safety and tolerability of VELNEZ in patients undergoing ear surgery in a surgeon’s routine clinical practice.

## Materials and methods

Ethical consideration

**Figure 1:**
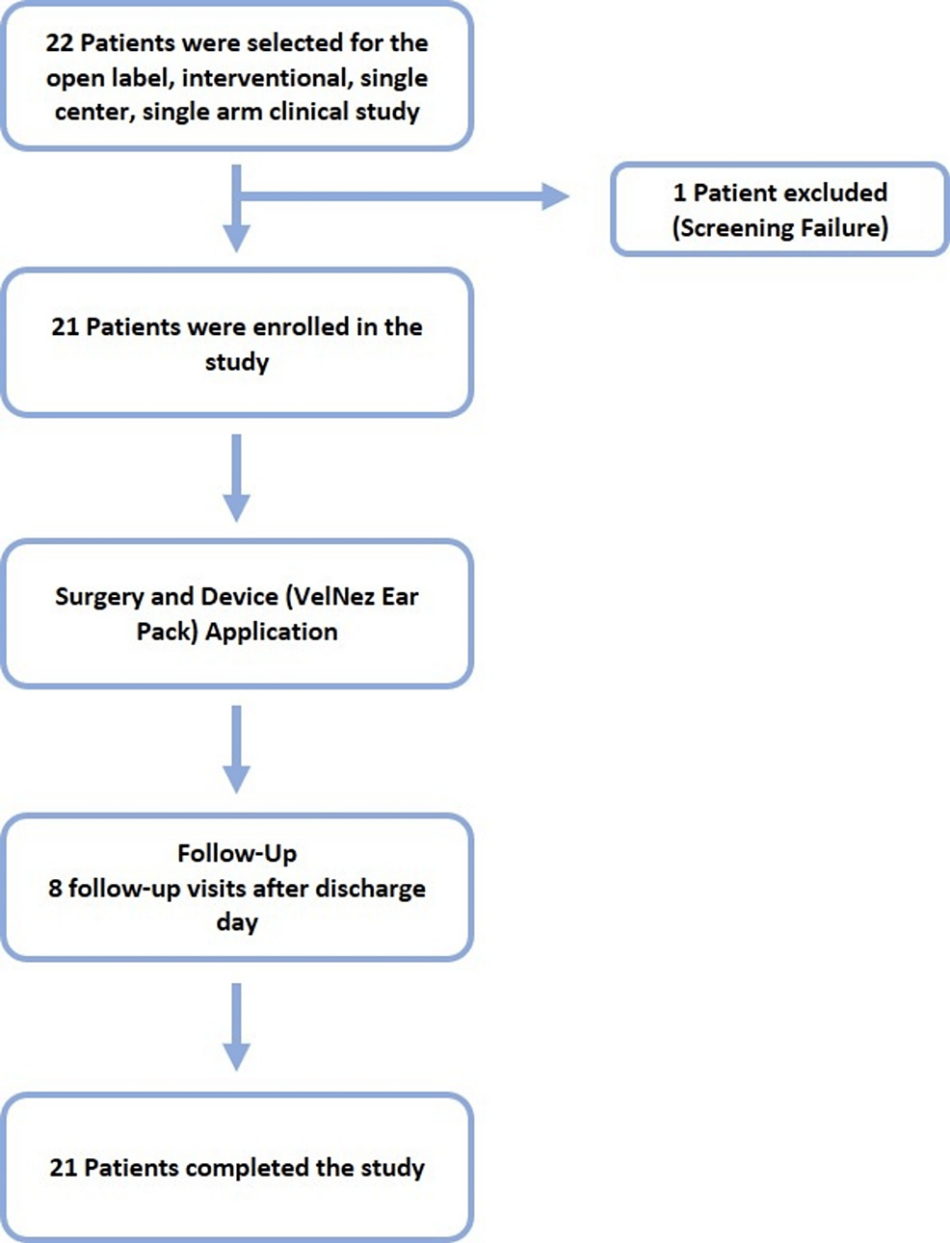
Flowchart of the study

Patients who gave informed written consent were recruited after obtaining approval from the Institutional Ethics Committee (EC registration number: ECR/1409/Inst/UP/2020) overseeing each site and Clinical Trials Registry- India (CTRI) registration (CTRI/2023/02/049434). Subject confidentiality was maintained throughout the study while obtaining data by participant-specific codes to maintain anonymity. The study was conducted in accordance with the International Conference on Harmonization Good Clinical Practice (ICH-GCP) guidelines, the Declaration of Helsinki, and the Indian Council of Medical Research (ICMR) guidelines (2017). This was an open-label, interventional, single-center, single-arm clinical study (Figure 1).

Participants and selection criteria

For this study, 21 subjects were enrolled. All potential subjects of both genders of 18-60 years of age, Asian only, and patients from all socioeconomic statuses who met the study-related inclusion and exclusion criteria were included. The inclusion criteria were (1) adults between 18 and 60 years of any sex, undergoing external and middle ear surgeries eligible to receive the ear plug in routine clinical practice; (2) subjects who can give informed consent in writing, and their data collected during study at predefined follow-ups; and (3) female subjects who were willing to take contraception for the entire duration of the study were included. The exclusion criteria were (1) patients who cannot be treated with ear plugs after planned ear surgery, cannot provide informed consent or have decision-making impairment, are positive for HIV, hepatitis B surface antigen (HBsAg), and hepatitis C virus (HCV), and unable or unwilling to comply with postoperative follow-ups; (2) subjects suffering from cholesteatoma or granulations in the middle ear, complicated chronic suppurative otitis media, having an active infection at the surgical site, and having a history of diabetes mellitus or hypertension; (3) subjects on aspirin, anti-platelet drugs, oral anticoagulants therapy, immunosuppression, corticosteroids, or chemotherapy; (4) pregnant females; (5) subjects who have a history of allergic (hypersensitive) reactions with any of the ingredients of the device, and concurrent participation in another clinical trial; and (6) subjects with a severe comorbid disorder, not expected to survive more than 12 months.

Surgical technique

Patients primarily underwent either tympanoplasty or mastoidectomy following standard surgical techniques. VELNEZ was minced into small pieces for placing inside the middle ear as a hemostatic and supporting agent for temporalis fascia graft. Likewise, VELNEZ was kept in the external auditory canal as a space-occupying dressing.

Study material

The study device, VELNEZ, is a composite sterile ear dressing. It was manufactured in a cleanroom of class 10,000 under a good manufacturing practice (GMP) certified manufacturing unit for standardized production and packing, which maintained the quality and stability of VELNEZ. The constituents of VELNEZ are gelatin, chitosan, polyvinyl alcohol, polyethylene glycol, and psyllium husk.

Imaging technique

MRI examinations were performed with a 1.5 T MRI scanner (Magnetom Sonata, Siemens, Erlangen, Germany) using a standard eight-channel head coil. To confirm the disintegration of the VELNEZ ear pack in the middle ear cavity, MRI images were obtained using axial and coronal T1, T2, short tau inversion recovery (STIR), and axial three-dimensional constructive interference in steady state (CISS) images, with non-echo planar imaging diffusion images. The disintegration was considered complete if fluid densities were seen in the middle ear.

Outcome measures

The patients in this study were evaluated for hemorrhage control along with patient comfort following the application of the VELNEZ ear plug on the day of surgery, discharge, and follow-ups. Tolerability and effectiveness of the ear plug were evaluated after ear surgery and weekly thereafter on all the follow-up visits based on the surgeon’s questionnaire (the surgeon rated the ease of using the device on a 1-5 rating on Likert scale, where 1 denoted “easy” and 5 denoted “difficult”) and monitoring of the adverse events. The questions were designed to evaluate the surgeons' experiences with the usage of VELNEZ (for example, ease of handling). The patient’s comfort level (pain, pressure, effect due to packing, infection, and general satisfaction) was assessed on a scale of 0 to 10, where 0 represents no/non-symptom and 10 represents severe symptoms. Assessment of non-adhesion and disintegration (using MRI) of VELNEZ in postoperative patients was also evaluated. This 60-day study consisted of eight follow‐ups after discharge. A telephonic follow-up was done with the enrolled subject by the investigator or authorized site personnel if the subject did not report on the scheduled visit.

Baseline parameters like medical history, universal medical assessment along with vital parameters, concomitant medication, urine pregnancy test for female subjects, and blood investigation (hematology and biochemistry), including serology, were assessed. Surgery site assessment was carried out for inflammation/swelling, itching, irritation, redness, pain (Visual Analog Scale (VAS) score), or bleeding event, and hemostasis assessments were recorded by the investigator on surgery day from the subject’s records.

A favorable endpoint was defined in terms of disintegration of the dressing/ear plug within 49 days, complete hemostasis within 10 minutes, reduction in pain and discomfort, and no sign of infection.

Statistical analysis

All statistical tests were performed using R Software (v4.3.0, R Foundation for Statistical Computing, Vienna, Austria). Normally distributed data were summarized as mean ± SD. Student's t-test was applied to analyze hematological and biochemical parameters. Statistical significance was taken at a 95% CI (p ≤ 0.05). Categorial data were summarized using counts and percentages along with a 95% CI for percentages.

## Results

Of the 21 subjects that were included, five were males and 16 were females, with ages ranging from 18 to 60 years (mean age: 28.4 years). No adverse event occurred to the patients during this study. Demographic data are shown in Table 1.

**Table 1:** Demographic characteristics of the study participants ^a ^Mean ± SD.

Patients enrolled	Age (years) (Min-Max)	Gender, N (%)		Height (cm) (Min-Max)	Weight (kg) (Min-Max)	Smoker, N (%)		BMI (kg/m^2^) (Min-Max)	Race, N (%)
		Male	Female			Yes	No		
21	28.4 ± 9.47^a^	5 (23.8)	16 (76.2)	160.7 ± 7.33^a^ (152-174)	59.2 ± 6.98^a^ (50-78)	0 (0)	21 (100%)	22.9 ± 1.31^a ^(21-26)	21 (100)

Hemorrhage control, one of the primary outcome measures, was evaluated within 10 minutes of surgery. Of the subjects, 100% had hemorrhage control within 90 seconds of surgery (Table 2).

**Table 2:** Hemorrhage control time: one of the primary outcome measures evaluated after VELNEZ application

Time	Subject population (%)	Number of patients
46-55 seconds	4.76	1
56-65 seconds	23.81	5
66-75 seconds	42.86	9
76-85 seconds	19.05	4
86-95 seconds	9.52	2

**Figure 2:**
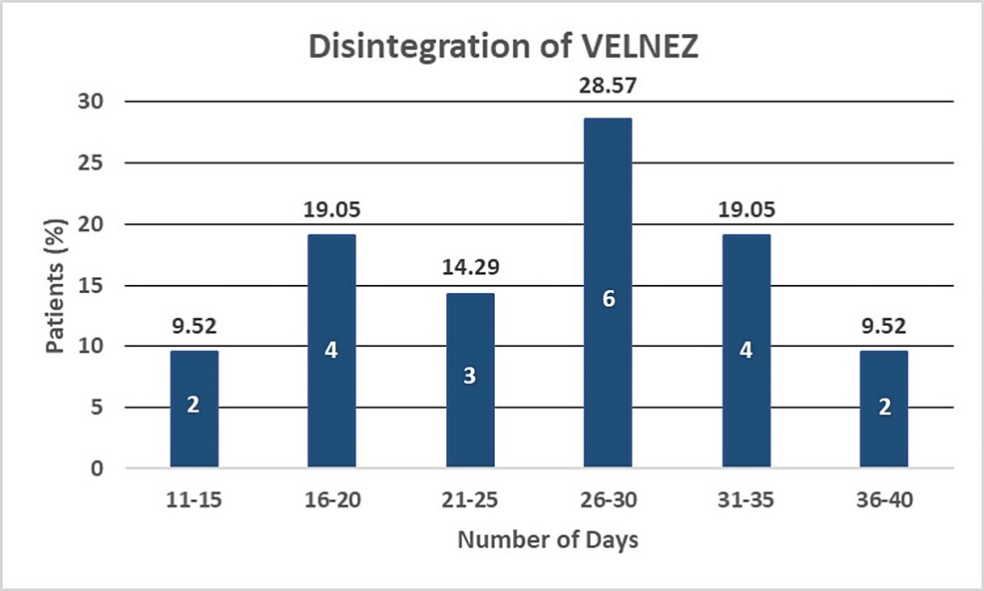
Disintegration of VELNEZ (percentage of population) The numbers inside the bar represent the number of patients within the range.

The average hemorrhage control timing was 1.08 ± 0.16 minutes. There was no patient data showing hemorrhage failure, i.e., hemorrhage control time >10 minutes. To evaluate the healing status data, follow-up otoscopic examinations were performed. Disintegration time was recorded when complete degradation of the ear pack took place (Figure 2).

The average time for achieving disintegration of the ear pack was 25.4 days. The effectiveness of VELNEZ was also evaluated by monitoring for infection during follow-up visits. All 21 (100%) subjects showed no infection on the day of discharge following surgery. Moreover, no migration or removal of the ear pack was observed over the complete course of the study.

Postoperative pain was evaluated through the pain VAS scale from surgery day to follow-up visit 10 within a duration of eight weeks. The subjects having moderate pain are summarized using counts and percentages along with a 95% CI for percentages by visit. Moderate pain is defined as 5 on the scale (VAS) of 1 (no pain) to 10 (worst pain). None of the subjects reported moderate pain from surgery day to follow-up visit 10 (day 51-60). Patients experiencing pressure in the ear canal due to ear pack application were evaluated. Of the subjects, 20 (95.2%) showed no pressure effect in the ear canal due to the application of the VELNEZ ear plug on the day of discharge. Also, no signs of infection at the site of the pack application were reported for any subject at any of the visits.

Global assessment of VELNEZ ear pack effectiveness was assessed by investigators based on the surgeon's questionnaire. A surgeons’ questionnaire was used to evaluate the use of the device. A 1-5 rating on the Likert scale was marked, where 1 denoted easy and 5 denoted difficult. Surgeon’s rating of VELNEZ, done on a Likert scale where 1 denotes easy and 5 denotes difficult, for various parameters is as recorded in Table 3.

**Table 3:** Surgeon’s questionnaire to evaluate the surgeons' experiences with the usage of VELNEZ Surgeons rated the device on a 1-5 rating on a Likert scale where 1 denoted “easy” and 5 denoted “difficult.”

Parameters	Response	Overall (N = 21), n (%)
Appropriateness of instruction for use	1	21 (100.0)
Conformance to tissue surfaces	1	21 (100.0)
Ease of application	1	21 (100.0)
Ease of handling	1	21 (100.0)

Blood samples were collected for hematological tests (Table 4), including RBC count, WBC count, and platelets count, and also for the measurement of cholesterol, glucose, and liver enzymes (Table 5).

**Table 4:** Mean value of hematological parameters of patients at screening day (visit 1) and last follow-up visit (visit 10) * p < 0.05; ^@^ biological reference range was defined from the raw data.

Parameter (unit)	Mean ± SD (Visit 1)	Mean ± SD (Visit 10)	Biological reference range (unit)^@^	p-value
Hemoglobin (gm/dl)	11.55 ± 1.38	11.83 ± 1.76	11.5-15 (gm/dl) (F), 13-17 (gm/dl) (M)	0.580
Total RBC count (million cells/mm³)	4.0 ± 0.51	4.04 ± 0.44	3.8-4.8 (million cells/mm³)	0.792
Total leucocyte count (TLC) (cell/mm³)	8072.72 ± 2783.48	7390 ± 2061.91	4000-10000 (cell/mm³)	0.375
Platelet count (lac cells/mm³)	1.85 ± 0.64	1.51 ± 0.39	1.50-4.50 (lac cells/mm³)	0.046*
Neutrophils (%)	61.77 ± 7.75	57.65 ± 7.56	40-80 (%)	0.089
Eosinophil (%)	2.86 ± 1.32	3.25 ± 0.85	1-6 (%)	0.271
Lymphocytes (%)	32.5 ± 7.55	35.6 ± 10.46	20-40 (%)	0.274
Monocytes (%)	2.86 ± 1.03	2.45 ± 0.75	2-10 (%)	0.151
Erythrocyte sedimentation rate (mm/hr)	19.18 ± 10.01	17.8 ± 6.16	Up to 20 (mm/hr) (F), up to 10 (mm/hr) (M)	0.597
Hematocrit (packed cell volume) (%)	31.91 ± 7.12	34.385 ± 5.10	36-46%	0.207
Mean corpuscular volume (MCV) (fl)	84.38 ± 6.66	80.945 ± 6.52	80-100 (fl)	0.099
Mean corpuscular hemoglobin (MCH) (picogram)	29.04 ± 2.55	31.036 ± 12.97	27-32 (picogram)	0.483
Mean corpuscular hemoglobin concentration (MCHC) (gm/dl)	34.40 ± 1.14	34.8505 ± 1.05	32-35 (gm/dl)	0.197
RBC distribution width (%)	14.54 ± 1.01	14.215 ± 1.03	11.5-14.5%	0.309

**Table 5:** Mean value of biochemical parameters monitored on screening day (visit 1) and last follow-up visit (visit 10) ^@^ Biological reference range was defined from the data available at the site.

Parameter (unit)	Mean ± SD (Visit 1)	Mean ± SD (Visit 10)	Biological reference range (unit)^ @^	p-value
Total bilirubin (mg/dl)	0.52 ± 0.16	0.44 ± 0.13	0.3-1.2 (mg/dl)	0.089
Alkaline phosphatase (IU/L)	209.79 ± 69.46	209.08 ± 51.85	60-290 (IU/L)	0.97
Serum glutamic pyruvic transaminase (IU/L)	34.81 ± 26.74	30.65 ± 11.65	5-42 (IU/L)	0.524
Serum glutamic oxaloacetic transaminase (IU/L)	32.25 ± 12.17	36.36 ± 16.08	5-40 (IU/L)	0.353
Creatinine (mg/dl)	0.82 ± 0.17	0.87 ± 0.19	0.4-1.4 mg/dl	0.453
Blood urea nitrogen, BUN (mg/dl)	11.08 ± 2.59	11.66 ± 4.35	7-20 (mg/dl)	0.594
Glucose, random (mg/dl)	88.95 ± 12.55	92.95 ± 30.17	70-140 (mg/dl)	0.573
Total cholesterol (mg/dl)	151.25 ± 26.49	148.45 ± 16.21	130-200 (mg/dl)	0.685
Glycosylated hemoglobin (%)	6.40 ± 0.65	6.20 ± 0.43	<6 non-diabetic level, <7 goal, >8 high risk	0.247

Samples were collected prior to surgery and at the last follow-up after intervention. The mean values were compared using Student's t-test, and results are presented as mean ± SD. No significant changes were observed for various parameters monitored; however, a significant decrease (p < 0.05) in platelet count was observed but the absolute platelet count was still well above normal limits.

## Discussion

Packing materials are commonly used in the middle ear and external auditory canal to help control the bleeding and provide support to the surgical site during the early stages of healing. A variety of ear packing materials have evolved over time. Ear packs are available as either absorbable or non-absorbable. The choice of ear packing material depends on the specific surgical procedure, the surgeon’s preference, and the patient’s needs. In the case of non-absorbable packing material, patients need a follow-up outpatient visit after surgery. In previous studies [7,8], non-absorbable packs were found to be very uncomfortable for patients postoperatively and painful to remove. To overcome the disadvantages associated with non‐absorbable packs, a variety of biodegradable ear packs have been developed [9-11]. Absorbable ear packing materials are effective as they accelerate the overall healing process and improve postoperative hearing [3]. Absorbable packs can also be easily shaped to fit the ear canal. Moreover, these can be impregnated with antibiotics or antimicrobial agents, which can help prevent infections. This study aimed to evaluate the clinical efficacy of a biodegradable ear pack in subjects undergoing planned ear surgery.

Bleeding management in the external auditory canal and the middle ear is challenging for surgeons, as it can obstruct the surgical field. The aspiration of the blood and debris needs to be removed for the progress of the operation. The main goals of ear packing are to provide support to compromised tissues, aeration of the middle ear cavity, and hemostasis [12]. The present study showed that VELNEZ was efficient in attaining hemostasis in a very short span of time. The effective hemostasis in the present study (within 90 seconds) could be attributed to chitosan, as in a previous study [13] where chitosan-based hemostatic bio-hydrogel was shown to demonstrate 1300% ± 50% blood absorbability. In yet another study [14], chitosan-polyvinyl alcohol sponges showed increased blood cell and platelet adhesion and activation, and improved blood clotting ability. In the same study, in vivo evaluations revealed excellent hemostatic performance and enhanced wound healing with decreased granulation tissue and increased re-epithelialization [14].

A patient experiences pain following ear surgery and on removal of the pack in case of non-absorbable ear packs. This study assessed the moderate pain using a VAS on surgery day, discharge day, and follow-up visits postoperation. No patient complained of moderate pain at any of the follow-up visits following surgery. In another study, comparing HA and gelatine-based absorbable packs, Deniz Hanci et al. [15] reported a gradual significant decline in pain levels over a duration of four weeks. In this same study, the patients were monitored for the requirement of painkillers (nonsteroidal anti-inflammatory drugs, NSAIDs), and none of the patients were on NSAIDs by the end of the fourth week. Pain alleviation is an important factor in deciding the tolerability of the ear pack. The present study results highlighted that VELNEZ successfully ameliorates moderate pain.

**Figure 3:**
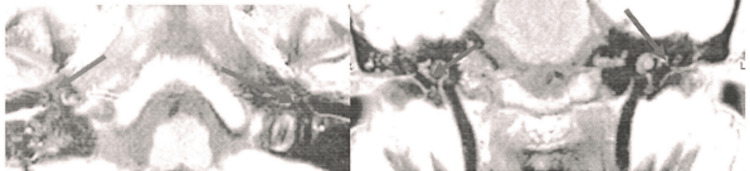
Representative MRI images of a treated patient indicating VELNEZ degradation The patient underwent right ear type I tympanoplasty. The right external auditory canal shows normal aeration. There is normal expected signal void in the right middle ear cavity and right mastoid antrum. The left tympanic membrane (neo-tympanum) is normal in appearance and there is no evidence of any residual soft tissue or fluid density in the middle ear.

In a previous study [16], Gelfoam packing after tympanoplasty resulted in delayed epithelization of the tympanic membrane (TM), severe fascia edema, and a larger air-borne gap due to an interrupted healing process of TM. In another study [9], extensive otorrhea was observed in patients following Gelfoam packing in cartilage graft myringoplasty procedures. Gelfoam usage can also result in extensive inflammation, adhesions, new bone formation, and fibrosis [4-6]. However, there are contrary studies showing no complications, no fibrosis, and favorable tissue properties associated with Gelfoam [12]. In the present study, no infection at the site of the VELNEZ application was reported in any of the patients under observation. Hematological tests showed no significant changes in erythrocytes and leukocyte counts. Biochemical parameters tests reveal normal functioning of the liver, as no significant changes were observed in serum glutamic pyruvic transaminase (SGPT) and serum glutamic oxaloacetic transaminase (SGOT) levels. Also, no changes were observed in total bilirubin, blood urea nitrogen, and alkaline phosphatase. In addition, MRI results showed complete disintegration of VELNEZ ranges from 12 days to 38 days from surgery day with average disintegration duration of 25.4 days (Figure 3).

The patient’s comfort was also evaluated by monitoring the pressure experienced due to the application of the VELNEZ ear pack. In a prospective randomized study [17], Gelfoam used in middle-ear packing caused more discomfort in patients during the early postoperative period in comparison to the group without packing. Zeitoun et al. [8] compared four different non-absorbable ear packs following middle ear surgery. The study concluded that none of these packs are superior and are associated with different side effects such as postoperative pain, discomfort, itching, pus and discharge, bleeding, and infection. Bismuth iodoform paraffin paste (BIPP)-impregnated gauze scored worst on most parameters and was the least satisfactory dressing in this study. In another study, Demir et al. [18] demonstrated absorbable ear packs composed of biodegradable synthetic polyurethane foam appeared to achieve better comfort to the patients in comparison to non-absorbable ear wick and ribbon gauze packs. In the present study, VELNEZ caused minimal discomfort as 95.2% of patients expressed no pressure in the ear canal on the discharge day. The ease of application and handling of the device, conformance to the mucosal surface of the ear, and its usage according to the instructions of use were analyzed using the surgeons’ questionnaire (Table 3). VELNEZ ear pack showed excellent results, scoring the lowest number on the 1-5 Likert scale, where 1 denotes easy and 5 denotes difficult for the above-mentioned parameters. There were no adverse events or serious adverse events reported in the study related to investigational products. Conclusively, VELNEZ can be considered a safe, efficient, and tolerable postoperative ear pack.

Non-absorbable packs such as ear wick, ribbon gauze (with bismuth, iodoform, paraffin paste, antibiotics, and antiseptic ointments), and silicone sheets need to be removed within seven to 21 days postoperatively. Removal is usually performed during a clinical visit without anesthesia [19]. The removal process of these packs can be uncomfortable [8], fairly painful [9], and may include risks of bleeding and graft displacement. These disadvantages can be overcome with the usage of absorbable pack. Absorbable packs improve patients' comfort as no secondary pack removal surgery is required, thus decreasing the risk of scarring and synechiae formation. In the present study, VELNEZ emerged as a safe and well-tolerable ear pack with no adverse events. In a previous study [20], Bellad et al. reported the safety, efficacy, and tolerability of VELNEZ as a nasal pack, and none of the subjects reported any adverse event or severe adverse event on using VELNEZ. Though the indication of the product was different, VELNEZ was found to be well-tolerable and safe. The observations are similar to the results obtained in the current study since no adverse events or reactions were noted in any manner whatsoever. This substantiates the compatibility of VELNEZ for human use irrespective of the indication against which it is being used.

Limitations

One of the major drawbacks of the study was that this was a single-arm study. The study outcome would have been more pronounced if a comparative group was included. Comparison of the product with existing commercial products or conventional methods will provide more information on the safety and efficacy of the product. However, the clinical insights from this present study were compared to existing research studies related to ear packs and associated complications. In addition, the number of patients included in the study was relatively small (n = 21). It will be desirable to know the efficacy and safety profile of the product on a larger number of patients. Another limitation of the study was that it allowed a short follow-up only, further studies showing the long-term effect following VELNEZ usage can provide insights on safety and tolerability.

## Conclusions

An ideal space-occupying ear dressing pack should control hemorrhage and secondary bleeding following ear surgery, accelerate the healing process, not cause infection, and provide comfort. The use of the VELNEZ pack after ear surgery fulfills these expectations. Results from the current study are very encouraging and it can be concluded that the VELNEZ ear pack is safe and effective and is therefore advisable in otology.
